# Clinical Presentation of Pediatric Celiac Disease Patients in the Qassim Region Over Recent Years

**DOI:** 10.7759/cureus.21001

**Published:** 2022-01-07

**Authors:** Resheed Alkhiari, Shahad M Aljameli, Dalal B Almotairi, Ghadah A AlHarbi, Layan ALmufadhi, Fatimah K Almeathem, Ali A Alharbi, Yasser AlObailan

**Affiliations:** 1 Medicine, College of Medicine, Qassim University, Buraydah, SAU; 2 Pediatrics, College of Medicine, Qassim University, Buraydah, SAU; 3 Pediatric Gastroenterology, Maternity and Children Hospital, Buraydah, SAU

**Keywords:** diabetes type 1, clinical manifestation, modified marsh criteria, pediatrics gastroenterology, celiac disease (ced)

## Abstract

Introduction

Celiac disease is an immune-mediated systemic disease. It is prevalent and has diverse clinical manifestations; gastrointestinal symptoms are more common in children, including failure to thrive, chronic diarrhea, vomiting, and abdominal distention. The diagnosis should be made at a precise time to evade severe irreversible complications, especially for pediatric patients. This study aimed to determine the clinical presentation and diagnosis, including laboratory, serological tests, and histopathological findings, in pediatric celiac disease patients.

Patients and methods

From January 2019 to August 2021, all children with a confirmed celiac disease diagnosis at Maternity and Children's Hospital in Buraydah, Qassim region, Saudi Arabia, were studied retrospectively. Information was collected, including demographics, clinical presentation, and diagnostic modalities with serology and small intestinal histology reported by Marsh grading.

Results

Fourteen patients were reviewed, with a mean age of 8.64 years. Marsh grading of those who underwent biopsy revealed that half of the patients had type 3a, and the rest had either type 1 or 3b celiac disease. Clinical manifestations included abdominal distention and chronic diarrhea, and some patients were asymptomatic.

Conclusion

Abdominal distention, chronic diarrhea, constipation, and nausea were the most common clinical features. Patients with a family history of celiac disease, longer symptom duration, and higher tissue transglutaminase immunoglobulin A* *(tTG-IgA) levels are more symptomatic.

## Introduction

Celiac disease (CD) is an immune-mediated systemic disease elicited by gluten in wheat and related prolamines from rye and barley in genetically susceptible individuals carrying human leukocyte antigen (HLA) markers DQ2 or DQ8 which cause damage to the small intestine [1,2]. CD presents diverse clinical manifestations; gastrointestinal symptoms are more common in children within two years of life, including failure to thrive, chronic diarrhea, vomiting, and abdominal distention. Furthermore, extraintestinal symptoms include iron deficiency anemia, osteoporosis, short stature, delayed puberty, in addition to many others [1]. Diagnosis of celiac disease is initially based on serology. For confirmation, an esophago-gastro-duodenoscopy with a duodenal biopsy was performed. Moreover, the treatment of CD is established by a strict, lifelong gluten-free diet [3].

Results from a study in Saudi Arabia that aimed to characterize the clinical presentations in children with celiac disease revealed that most children had non-classical presentations or were part of high-risk groups for celiac disease [4]. Studies show that anemia is the most common presenting symptom in children, followed by diarrhea [5]. Another retrospective study confirms that the classic presentation is found predominantly in children, and they most commonly present with diarrhea [6]. Furthermore, a study in India evaluated Indian children with CD and compared them to children in the west; the results showed a more significant number of children presenting with classic features along the lines of diarrhea, failure to thrive, and anemia [7]. Another review of CD presentations in the UK demonstrates that the most common gastrointestinal symptoms were abdominal pain, diarrhea, and bloating. A report showed low ferritin, a moderate to severe vitamin D deficiency, and anemia as extraintestinal signs [8]. Chronic diarrhea was followed by failure to thrive in the classical type, whereas short stature was the most frequent feature observed in non-classical CD [9].

Gender, age, and demographics are strongly associated with the prevalence of celiac disease. Previous studies have shown female predominance in both children and adults [10-12]. A global systematic review estimated that the highest prevalence of the disease was in Europe and the least was in South America [10]. Another study among the adult population showed that the highest prevalence of CD was seen in Saudi Arabia and the lowest in Tunisia [12]. In Saudi Arabia, a meta-analysis estimated that the prevalence of confirmed CD ranges between 1.4% and 2.7% [13]. Another study among different Saudi regions revealed that the seroprevalence of CD is highest in Al-Qassim [14]. Other autoimmune conditions such as type 1 diabetes, autoimmune thyroid disease, juvenile arthritis, and selective IgA deficiency may be associated with an increased or higher risk of the disease severity and complications [15-17].

Since celiac disease is prevalent and has various clinical presentations, the diagnosis should be made at a precise time to evade severe irreversible complications, especially for pediatric patients [3,4]. This study aims to determine the clinical presentation of pediatric celiac disease patients in the Qassim region and identify the risks associated with the disease.

## Materials and methods

Study design, area, population, and sampling

This is an observational retrospective cohort study conducted at the Department of Gastroenterology and Hepatology, Maternity and Children's Hospital (MCH), Buraydah. All pediatric patients up to 16 years old from 2019 to 2021 who had a confirmed celiac disease diagnosis with serology and intestinal biopsy were included in the study. Patients with incomplete medical records and adult patients were excluded from the study.

Methods for data collection 

We retrospectively collected our data from patients’ medical records that fit our inclusion criteria from January 2019 to August 2021. The needed data were entered in an excel sheet and saved on one secured, password-protected laptop to ensure data safety. We obtained approval from the Qassim Regional Ethics Committee (NCBE, No. H-04-Q-001) for the application and publication of the study (approval number 1443-598061).

Statistical analysis

The software SPSS Statistics version 26.0 (IBM Corp., Armonk, NY, USA) was used to analyze the data. The descriptive statistics were demonstrated by utilizing means, standard deviations, numbers, and percentages whenever appropriate. The measures of central tendency of the VHI score have been performed using the Shapiro-Wilk test. The relationship between celiac disease and patient characteristics has been performed using the Fischer exact test and the Mann-Whitney test. The significance level adopted was 5% (P < 0.05).

## Results

We reviewed 14 pediatric patients with CD. As seen in Table 1, the mean age of the patients was 8.64 (SD 3.84) years, with the majority being females (71.4%). Consanguinity was identified in one case, three cases with a family history of CD, and another three with a family history of type 1 diabetes. Marsh grading of those who underwent biopsy revealed that seven patients had type 3a, four had type 3b, and only one patient came with a type 1 CD. The most common associated condition was type 1 diabetes (35.7%). 

**Table 1:** Patient history and characteristics

Variable	N (%)
Age in years (mean ± SD)	8.64 ± 3.84
Gender	
Male	04 (28.6%)
Female	10 (71.4%)
Consanguinity	
Yes	01 (07.1%)
No	02 (14.3%)
Unknown	11 (78.6%)
Family history of celiac disease	
Yes	03 (21.4%)
No	06 (42.9%)
Unknown	05 (35.7%)
Family history of type 1 DM	
Yes	03 (21.4%)
No	04 (28.6%)
Unknown	07 (50.0%)
Clinical features	
Asymptomatic	04 (28.6%)
Abdominal distention and chronic diarrhea	08 (57.1%)
Constipation and nausea	02 (14.3%)
Marsh grading	
Type 1	03 (21.4%)
3a	07 (50.0%)
3b	04 (28.6%)
Associated condition	
None	07 (50.0%)
Type 1 diabetes	05 (35.7%)
Epilepsy	01 (07.1%)
Thyroid disease	01 (07.1%)

Clinical profiles and laboratory parameters are shown in Table 2. The patients’ mean height was 120.7 cm, while their mean weight was 30.7 kg. The mean age at presentation was 6.86 years, while the mean duration of symptoms was 17.1 months. In addition, the mean values of hemoglobin, vitamin D level, calcium level, alkaline phosphatase level, aspartate transaminase (AST), alanine aminotransferase (ALT), albumin, and tissue transglutaminase immunoglobulin A (tTG-IgA) were 11.4, 20.4, 3.19, 254.1, 25.7, 16.3, 40.3, and 442.7, respectively.

**Table 2:** Patients' clinical profiles and laboratory parameters

Variable	Mean ± SD
Height (cm)	120.7 ± 22.9
Weight (kg)	30.7 ± 14.3
Age at presentation	6.86 ± 3.86
Duration of symptoms in months	17.1 ± 17.8
Hemoglobin level	11.4 ± 2.18
Vitamin D level	20.4 ± 9.12
Calcium level	3.19 ± 2.53
Alkaline phosphatase level	254.1 ± 50.6
AST	25.7 ± 11.5
ALT	16.3 ± 9.49
Albumin	40.3 ± 3.29
tTG-IgA	442.7 ± 619.1

When measuring the prevalence of celiac disease in relation to the characteristics of the patients at the presentation, it was found that the prevalence of symptomatic patients was more common among those with a family history of CD (p=0.010; Table 3).

**Table 3:** Relationship between celiac disease among the patient characteristics and clinical manifestation (n=14) ^§^P-value has been calculated using the Fischer exact test. **Significant at p<0.05 level.

Factor	Celiac disease		P-value^§^
	Asymptomatic	Symptomatic	
Age in years (mean ± SD)	9.50 ± 1.91	8.30 ± 4.42	0.945
Gender			
Male	01 (25.0%)	03 (30.0%)	1.000
Female	03 (75.0%)	07 (70.0%)	
Family history of celiac disease			
Yes	0	03 (30.0%)	0.010**
No	0	06 (60.0%)	
Unknown	04 (100%)	01 (10.0%)	
Family history of type 1 DM			
Yes	01 (25.0%)	02 (20.0%)	0.357
No	0	04 (40.0%)	
Unknown	03 (75.0%)	04 (40.0%)	
Associated condition			
Yes	03 (75.0%)	04 (40.0%)	0.559
No	01 (25.0%)	06 (60.0%)	

We also observed that the mean duration of symptoms was statistically significantly longer in symptomatic patients (p=0.010), while the mean value of tTG-IgA in symptomatic patients was significantly higher (p=0.033). On the other hand, the mean weight of asymptomatic patients was statistically significantly higher than that of symptomatic patients (p=0.047). Other clinical manifestations of the patients did not significantly differ between asymptomatic and symptomatic patients (p>0.05; Table 4).

**Table 4:** Relationship between celiac disease in regards to the patients' clinical profiles and laboratory parameters (n=14) ^‡^P-value has been calculated using the Mann-Whitney test. **Significant at p<0.05 level.

Factor	Mean ± SD (asymptomatic)	Mean ± SD (symptomatic)	P-value^‡^
Height (cm)	130.0 ± 0.00	118.7 ± 25.2	0.582
Weight (kg)	42.0 ± 10.9	26.1 ± 13.3	0.047**
Age at presentation	8.75 ± 1.71	6.10 ± 4.28	0.374
Duration of symptoms in months	0.50 ± 0.71	20.4 ± 17.7	0.030**
Hemoglobin level (g/dL)	12.6 ± 1.42	10.9 ± 2.31	0.260
Vitamin D level (ng/mL)	22.8 ± 8.64	18.1 ± 10.2	0.686
Calcium level (mg/dL)	2.43 ± 0.05	3.62 ± 3.17	0.788
Alkaline phosphatase level (U/L)	261.5 ± 19.2	246.7 ± 75.2	1.000
AST (U/L)	18.7 ± 4.51	31.0 ± 12.9	0.229
ALT (U/L)	9.00 ± 2.65	21.7 ± 9.11	0.229
Albumin (g/L)	39.5 ± 2.12	40.5 ± 3.66	0.667
tTG-IgA	110.7 ± 92.6	585.0 ± 702.4	0.033**

## Discussion

The present study was carried out to examine the clinical manifestations of pediatric patients with CD. Studies suggest that the predominant symptom of CD is chronic diarrhea [5,7,9]. The most common symptoms were abdominal distention and chronic diarrhea among the ten symptomatic patients in this study. These findings are consistent with the paper by Oliveira et al. [8]. They reported that 60% of pediatric patients had a classical type of CD, 25% had non-classical, 10% were subclinical, and 5% were classified as potential CD patients. The most frequent clinical features were abdominal pain (58%), diarrhea (43%), and bloating (27%). This has been supported by Riznik et al. [18]. Accordingly, they found out that the most common symptoms of symptomatic children were abdominal pain, growth retardation, and diarrhea. In Bahrain [2], pallor and failure to thrive were the most common features at the presentation, while in Riyadh, Saudi Arabia [4], failure to thrive was a major symptom at the presentation, followed by short stature.

Furthermore, in our study, the majority of the pediatric patients were females (71.4%), with no difference between symptomatic and asymptomatic CD. A similar proportion of CD cases has been reported by Imran et al. [9], where females have CD more than males, which is also consistent with the paper of Dehbozorgi et al. [19]. On the other hand, Isa et al. [2], as well as Saeed et al. [4], documented that CD male patients were more than CD female patients.

A positive family history of CD had been detected in three cases, all of them being symptomatic patients. In Bahrain [2], findings indicated that 30.2% (n=13) had a family history of CD with a similar proportion (24%) as reported in Portugal [8]. Moreover, the most commonly associated disease was type 1 diabetes, which was consistent with the papers of Saeed et al. [4] and Dehbozorgi et al. [19]. However, in a study done by Isa et al. [2], iron deficiency anemia (69.7%) and history of food allergy (25.4%) were the most common underlying diseases of children with CD.

Based on biopsy, half of these patients (50%; n=7) had a Marsh grading score of 3a. In Portugal [8], 94% of patients had modified Marsh 2 or 3 enteropathy, while the rest of the patients had normal histology with positive human leukocyte antigen typing. In Iran [19], the histology of patients was classified into Marsh 3a (33.3%), Marsh 3b (32.4%), and Marsh 3c (29.5%).

Our results further noted that symptomatic patients had a significantly longer duration of symptoms and higher mean values of tTG-IgA but significantly lower by weight. A significant proportion of positive anti-tTG has also been reported by Saeed et al. [4]. Based on their accounts, a positive anti-tTG had been detected in 91.5%, but there was no difference in titers between classical and non-classical CD. In another study conducted by Imran et al. [9], they found that the tTG-IgA titer was significantly higher in 80% of the CD patients, which was also comparable with our results.

The study limitations for this current study include a lower number of patients and patients only up to age 16 were included in our study, as other older children were referred to other hospitals. 

## Conclusions

Most of the pediatric patients were symptomatic. Abdominal distention, chronic diarrhea, constipation, and nausea were the most commonly reported clinical features. Patients with a family history of CD, who had longer symptoms duration, and who had higher tTG-IgA values tend to be more symptomatic. Type I diabetes was the most common associated disease. Further research is needed to get more insights into the spectrum of this disease. A future multicenter study is recommended to obtain a better assessment of the clinical manifestations of Saudi children with CD.
